# Reflections on the current context and assessment of undergraduate medical education in Brazil

**DOI:** 10.1590/0100-6991e-20243857-en

**Published:** 2024-12-12

**Authors:** GERSON ALVES PEREIRA, RAMIRO COLLEONI, ROSANA ALVES, HERMILA TAVARES VILAR GUEDES, JORGE CARVALHO GUEDES, PEDRO TADAO HAMAMOTO, JOSÉ EDUARDO LUTAIF DOLCI, CESAR EDUARDO FERNANDES

**Affiliations:** 1 - Universidade de São Paulo, Campus Bauru, SP, Brasil. Presidente da Comissão do Título de Especialista do Colégio Brasileiro de Cirurgiões.; 2 - Universidade Federal de São Paulo, SP, Brasil. Presidente da Comissão de Residência Médica do Colégio Brasileiro de Cirurgiões.; 3 - Centro Universitário FAESA, Vitória, ES, Brasil. Instituto Capixaba de Ensino, Pesquisa e Inovação.; 4 - Universidade Estadual da Bahia, Salvador, Brasil.; 5 - Universidade Federal da Bahia, Salvador, Brasil. Coordenador da Comissão do Título de Especialista da Federação Brasileira de Gastroenterologia; 6 - Universidade Estadual de São Paulo, Campus Botucatu, SP. Núcleo Interinstitucional de Estudos e Práticas de Avaliação em Ensino Médico; 7 - Faculdade de Ciências Médicas da Santa Casa de São Paulo, Brasil. Diretor Científico da Associação Médica Brasileira (AMB); 8 - Faculdade de Medicina do ABC, Santo André, SP, Brasil. Presidente da Associação Médica Brasileira (AMB).

**Keywords:** Training of Human Resources in Health, General Practitioners, Licensure in Medicine, Capacitação de Recursos Humanos em Saúde, Clínicos Gerais, Licenciamento em Medicina

## Abstract

This reflection article can be divided into two complementary parts. The first shows the current context of medical education in Brazil, bringing the most recent data on the number of undergraduate students and its growing gap in relation to the reduced number of vacancies in medical residency, which, despite being the most appropriate modality to train specialists, has been overlooked by the excessive number of lato sensu graduate courses. The second part discusses the possibilities of evaluating medical training, which is terminal in Brazil, enabling the newly graduated physician to claim the professional registration and to practice medicine, being evaluated only by their own medical course. It also reviews the historical evolution and pros and cons of evaluating medical education for doctors trained both locally and abroad.

## RESUMO

Este artigo de reflexão pode ser dividido em duas partes complementares. A primeira mostra o contexto atual da formação médica no Brasil trazendo os dados mais recentes do número de graduandos e sua crescente defasagem em relação ao número reduzido de vagas de residência médica que, mesmo sendo a modalidade mais adequada para formar especialistas, tem sido preterida ao número excessivo de cursos de pós-graduação Lato Sensu. A segunda parte discute as possibilidades de avaliação da formação médica, que é terminal no Brasil, possibilitando que o médico recém-formado possa ter o registro profissional para exercer a medicina, sendo avaliado apenas pelo seu próprio curso médico. A evolução histórica das iniciativas de avaliação da formação médica é mostrada e discutida em termos técnicos acerca dos prós e contras, envolvendo os médicos formados no Brasil e no exterior.

## INTRODUCTION

The undergraduate degree in Medicine in Brazil is characterized by terminality, which allows the recent graduate full license to practice Medicine by completing the course in one of the country’s medical schools. 

Upon graduating, usually after six years, doctors registered with the Regional Council of Medicine (CRM) can work in offices, clinics, and hospitals. However, they will function as generalists. 

Although it is not mandatory for the practice of the profession, residency is considered the “gold standard” of medical specialization (BRASIL, 1981). Residency programs grant the title of specialist. The other way to obtain a specialist title is by meeting the criteria to take the title exams of any medical society associated with the Brazilian Medical Association (AMB). These are the two types of titles accepted by law (BRASIL, 2015) for a doctor to obtain the Specialist Qualification Registration (RQE), a document that proves that a doctor is qualified to work in a medical specialty.

There are already more than 210,000 general practitioners in Brazil among the 575,930 active physicians (CFM, 2024), one of the largest physician populations in the world, in an accelerated evolution. The term “generalist” designates a doctor without a specialist title, with general training in medicine. The National Curriculum Guidelines for the Undergraduate Course in Medicine (CNE Resolution No. 3/2014) emphasize that the graduate will have general training (art. 3), that the undergraduate degree in medicine aims at the training of general practitioners (art. 6) and professionals with a generalist profile (art. 29). The Brazilian Classification of Occupations (CBO) does not assign specialty to general practitioners - code 2251-70 (SCHEFFER et al, 2023). 

Thus, we can consider that medical graduates in the country are generalists, although there is practically a consensus that the six years of graduation do not guarantee the proposed terminality, with the ability to solve 80% of health problems, and there is a proposal that medical residency be mandatory for all graduates. This is one of the crucial reasons justifying the evaluation of medical graduates.

In First World countries, it is mandatory to complete medical residency for the certification of professional practice. That is, there is a concern with doctors specializing, whether through public or private means, in a program that has the tutoring and applied knowledge of a professional with more experience in that area of knowledge. In all cases, there is also immersion of the professional in environments where the health service is offered. In fact, the quality checklist of training conducted by countries always considers characteristics about the environment in which physicians learn about their specialization. These findings reveal how valuable it is to enable this form of training for physicians (ENAP, 2021).

Monitoring trends and understanding motivations are essential to better understand the panorama of medical education in Brazil.

This large and growing contingent of general practitioners has multiplied the Lato Sensu Post-graduate Courses (PGLS), also deemed “Specialization Courses”, defined by the Ministry of Education (MEC) as continuing education programs aimed at those who have already completed their undergraduate studies, aiming at complementary academic training and professional updating in the various fields of knowledge (BRASIL, 2018). A recent study identified 2,148 PGLS courses in medicine offered by 373 institutions. About 40% of them operate in the distance learning (DL) modality, more than 90% are paid, marketed by private institutions, and most (60%) concentrate in the States of São Paulo, Minas Gerais, and Rio de Janeiro. Endocrinology, Dermatology, Psychiatry, and Radiology are the most common specialties (DIAS et al, 2024). 

There are PGLS maintained by renowned HEIs or accepted by specialty societies in the scores of title tests. But many others are offered by institutions without experience or capacity in the course area. It is also important to highlight that some institutions outsource courses execution. In view of this, there are worrisome moves to make specialized medical training more flexible or to consider PGLS courses as alternatives to Medical Residency (MR) and to titling via Specialty Societies, which should be reinforced as appropriate modalities of medical specialization (DIAS et al, 2024).

The Brazilian Medical Association (AMB) has denounced what it calls “specialization courses outside Brazilian legislation” (AMB, 2024a). The National Council of Education (CNE), the MEC and the CNRM explained the singularities and the normative framework that differentiate MR from PGLS (AMB, 2024b).

The 2023 Medical Demography panel demonstrated the exacerbation of the funnel effect formed by the disproportionate growth of undergraduate vacancies in relation to MR vacancies. That study found 389 medical schools that, together, offered 41,805 undergraduate vacancies (77% in private courses).

**Figure 1:**
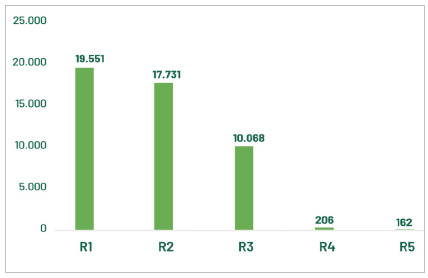
Resident physicians in Brazil, according to the current MR year (R1 to R5), in 2024. Source: CNRM/Sesu/MEC; Scheffer, M. et al. Medical Demography in Brazil. Note: R1 vacancies include those with direct access and those requiring prerequisites.

The most current panorama showed that 47,700 doctors were attending MR in 2024. Those resident physicians represented about 8% of the total number of physicians in the country. A total of 19,551 physicians were enrolled in R1, the first year of Medical Residency. The number of accredited Medical Residency Programs (MRP) was 5,631 in 2024, presented in the 55 specialties and 62 recognized areas of activity (BRASIL, 2024a), recognized by the Joint Committee on Specialties (CME), composed of representatives of the National Commission for Medical Residency (CNRM), the Federal Council of Medicine (CFM) and the Brazilian Medical Association (AMB) (SCHEFFER et al, 2024). Figure 1 shows the general distribution of resident physicians categorized by current period (year) in the Medical Residency Programs in 2024.

At the end of 2023, another 10,000 new undergraduate vacancies in medicine were authorized by the Ministry of Education, which were not computed in the Medical Demography panel of the same year and will make the bottleneck effect even greater for MR vacancies soon.

**Figure 2:**
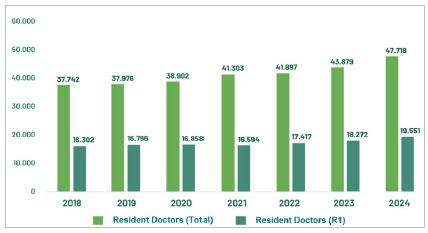
Evolution of the total number of physicians attending medical residency and physicians in the first year (R1), from 2018 to 2024. Source: CNRM/Sesu/MEC; Scheffer, M. et al. Medical Demography in Brazil. Note: R1 vacancies include those with direct access and those requiring prerequisites.

To great surprise, contrary to what previously happened, when the vast majority of graduates were interested in attending a MRP, even with the large increase in undergraduate vacancies throughout the country, there has been a decrease in the demand for vacancies in R1 annually, as can be seen in Figure 2.

**Figure 3:**
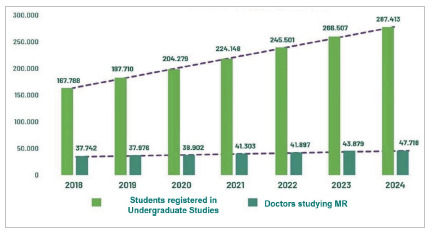
Evolution of the number of students enrolled in medical schools and resident physicians attending the first year (R1), from 2018 to 2024.

Source: CNRM/Sesu/MEC; INEP/MEC; Scheffer, M. et al. Medical Demography in Brazil. Note: *For the calculation of students enrolled in undergraduate courses in 2024, an estimate was made based on previous years of the Inep Census.

Figure 3 shows that from 2018 to 2024, the gap between undergraduate medical school enrollments and medical residency vacancies is widening significantly, unless there is an increase in the supply of MRPs.

The decision to postpone MR may be a valid choice for some graduates, allowing them to explore different opportunities and make better-informed decisions about their medical careers. Medical students’ decisions can be influenced by a wide variety of factors, such as:

1. MRP Challenges: The perception of challenges associated with Medical Residency, such as high and dense workload, stress, and lack of work-life balance can lead some graduates to postpone their entry into an MRP. This perception was clear during and after the SARS-CoV-2 pandemic.
2. Difficulties in Accessing Residency: In some cases, fierce competition and limited places in MRPs can make access difficult, leading graduates to consider other options.
3. Choose Other Professional Pathways - such as research, teaching, health management, public health, or sectors not related to clinical practice.
4. Desire to Explore Different Areas of Medicine - gain experience in different areas of medicine before deciding on a specific specialty. This may include working in different care settings, such as emergency units, primary care, or in different clinical specialties.
5. Need for Time to Make Decisions - need for additional time to evaluate preferences, goals, and interests before committing to a Medical Residency.
6. Search for International Experience - exchange programs or MRP in other countries, which can enrich skills and professional prospects.
7. Economic Scenario and Labor Market - economic conditions, especially of those graduates with debts accumulated in FIES and in the financing to pay for private courses, in addition to the labor market for health professionals without specialization, especially when they offer attractive remuneration, can influence the decision of newly graduated physicians not to apply for MRP, even aware that, in many cases, there is no job security.
8. Flexibility in Medical Careers - changes in legislation or in the structure of MRP that allow greater flexibility in medical careers, providing different options for training or professional performance.
9. Preference for Other Graduate Modalities - opt for improvement courses in specific areas, mistakenly self-named “Specialization”; or even for Stricto Sensu Graduate Programs (master’s, doctorate).
10. Personal and Family Issues - personal considerations, such as the need to spend more time with family, personal health issues, or other family factors, can also influence the decision not to attend an MRP.

These factors may vary among graduates and reflect individual circumstances and preferences. Such reasons are multifactorial and can vary between individuals. In addition, trends can change over time in response to evolutions in the healthcare system, government policies, and other external influences. 

### Evaluation of medical training at the undergraduate level

Since the mid-1980s, several scholars have questioned the terminality of undergraduate medical education and have shown a greater interest in evaluating its quality (YAZLLE ROCHA, 1983; GONÇALVES, 1986; STELLA et al, 1997). Particularly at the beginning of this century, such questioning has increased, with the discussion about the introduction of a licensing exam to assess the aptitude of undergraduates to practice. Since then, four Legislative Bills (LB) have been proposed: LB 840/2003, LB 217/2004, LB 559/2007, and LB 165/2017. Meanwhile, other sectors of the national medical movement argue that the evaluation should be continuous, including the analysis of the teaching conditions offered, with a close look at the quality of the schools’ infrastructure and teaching staff (COLLUCCI apud BICA & KORNIS, 2021; CFM, 2017a). Thus, to obtain medical registration and be practice authorization, it would no longer be sufficient to complete the undergraduate course. 

Among the reasons for a broader discussion about the quality of medical training are the increase in the number of medical courses and new vacancies in courses already in operation. Another reason is that, with greater access to communication spaces, there has been greater dissemination of medical errors and structural failures in health systems. So, the increase in global demand for quality assurances in the provision of medical services has been a result of a public awareness of such problems, as well as their right to better services. This is reflected in the increase in complaints about alleged malpractice in the Regional Medical Councils, and in lawsuits involving medical and health services. In Brazil, such growth has been explosive: between 2010 and 2014, there was a 140% increase in the number of cases that reached the Superior Court of Justice on an appeal basis (CAMBRICOLI, 2015 apud BICA & KORNIS, 2021). 

These events have been pressuring the medical community to demand quality guarantees from universities and the State in the training of professionals and, sometimes, to try and take control over their evaluation processes. In the Brazilian case, it can be argued that the requirement of a “Guarantee Seal” for professionals, within this more consumerist logic, also falls on the representatives of the private supplementary health system that serves the layers with higher purchasing power (SCHEFFER, 2015).

This trend also reflects the current increased focus on “patient safety” and “public trust” in the protocols of international medical service quality certification systems. On the other hand, the interference of the Brazilian State (MEC, National Council of Education - CNE, National Council of Health - CNS, among other bodies) in the curricular design of medical education, materialized through the National Curriculum Guidelines (DCNs) of 2001 and 2014, and the current discussion of its updating, also represents a response to these social demands (BRASIL - MEC, 2024). 

There are also other important factors involved, like the greater international mobility of the medical workforce, making it inevitable that it will be composed in large part of professionals whose training one has little management over or knowledge about, and the global increase in the number of medical schools, a phenomenon that generates, also in the international academic community, the perception that the quality of education offered by the new schools may not be sufficient for the adequate care society craves. 

Together, these factors strengthen the arguments in favor of the introduction of Medical Licensing Examinations (MLE) as instruments of external regulation, a phenomenon that has recently occurred in countries such as Germany and Switzerland, and is ongoing in the United Kingdom, where a national medical entity (the General Medical Council) has decided in favor of its implementation as of 2023 (SWANSON & ROBERTS, 2016).

In the Brazilian case, causes for the recent interest on a national medical learning evaluation are the definition, by the 1988 Constitution, of the Public Unified Health System (SUS) as the organizer of the training of health professionals and the vigorous expansion, since then, of the population’s access to primary care through the family health strategy (PAIM et al., 2011). As a result, linking medical education to the demands of the labor market became a formal objective of the Brazilian State, indicating that it could become the object of greater external regulation - which was made effective by the elaboration, in 2001 and 2014, of the National Curriculum Guidelines (DCNs) for medical courses - and of new forms of evaluation (BICA & KORNIS, 2021). 

The general aptitude of a medical student is understood by educators and professional councils as the satisfactory performance in well-defined competencies that, together, unite the dimensions of a safe and qualified practice. Such competencies include theoretical knowledge, psychomotor skills, interpersonal communication, professional ethics, and clinical reasoning applied to situations in medical practice (EPSTEIN & HUNDERT, 2002). 

The employment of the competency-based model implies the implementation of measures by evaluation methods. “Knowing” and “knowing how” are domains of cognitive competences, which is the professional’s theoretical knowledge. The following domains, on the other hand, bring together the competencies related to the practical application of the acquired knowledge. “Show how” indicates what the professionals can do and allows one to know their clinical skills. This includes psychomotor (physical examination, medical procedures, etc.) and behavioral (communicating with patients and colleagues, complying with patient safety procedures, etc.) competencies. “Doing”, on the other hand, describes the real physicians’ performance, that is, how they really perform they functions in the midst of the pressures of a dynamic environment, with its uncertainties and subjectivities, and shows their ethical behavior in the face of emotionally complex situations and flexibility in the face of diverse demands (BOURSICOT et al, 2011). 

The most frequently presented option is the so-called “Bar Exam”, which is an evaluation of medical training at the end of the course, as occurs in the Brazilian Attorneys Bar Association (OAB), whose approval would be necessary to obtain registration with the Medicine State and Federal Councils. The international experiences accumulated about these MLEs clearly show that, with the methods currently available, the assessment of a recent graduate’s aptitude to practice medicine is not effective (BICA & KORNIS, 2020). The idea presented that such an examination would be able to solve all problems related to medical malpractice is not justified. After all, even the most sophisticated test models do not assess performance, skills, and attitudes in the workplace - an essential dimension of the aptitude in question. 

The objective of the MLE is to identify candidates who can practice medicine. To this end, it is necessary to use methods that can assess, with acceptable accuracy, the competencies necessary for a safe and qualified practice (BICA & KORNIS, 2020).

MLEs are a type of summative assessment to certify the individual or to define their aptitude to progress to the next stage of their academic or professional career. Such evaluations are described as “high stakes” due to the consequences they imply for the individual’s future (BICA & KORNIS, 2020).

Summative assessments do not seek to interfere with the individual’s learning - when they do, it is an unintended effect. On the other hand, formative assessments have precisely this objective: to measure the individual’s proficiency in certain competencies to define learning objectives and allow changes in an educational program. Therefore, formative and summative evaluations are not antagonistic, but complementary.

The greatest formative assessment experience, aimed at evaluating and giving feedback on the performance of students and schools, is the Progress Test (PT) during graduation. Internationally conceived from concomitant initiatives in Canada and the Netherlands (VAN DER VLEUTEN, 1996; FREEMAN, 2010), the PT consists of multiple-choice tests, applied in a serial manner throughout the undergraduate course. To ensure its effectiveness, the PT is based on a comprehensive content matrix, which includes topics of epidemiological relevance, regional adaptations, distinct stages of the life cycle, and ethnic and social diversity. When correctly elaborated, applied, and interpreted, PT offers detailed feedback to students and schools, helping to identify effective areas of the curriculum, and allowing non-ranking comparison of institutions. It also allows the early detection of students with insufficient performance and the individual perception of each student as to their evolutionary performance in knowledge.

Implemented in Brazil in 1998 (SAKAI et al, 2008) and made a national project with the implementation of interinstitutional centers from 2014 (BICUDO, 2019), the PT sought to guarantee the internationally recognized basic principles (VAN DER VLEUTEN, 1996), aiming at collaborative networking and teacher development from the development of regional consortia, congregated to apply the exams. The expansion of the PT scope to the national level, based on the initiative of the Brazilian Association of Medical Schools (ABEM), boosted the PT as one of the alternatives for the national evaluation of schools and graduates.

In 2018, the National Meeting of Medical Entities released a favorable position for the serial exam, with the justification that the medical course involves teaching characteristics of cognitive, psychomotor, and behavioral aspects that should not be evaluated in a single process, but in a continuous way throughout the undergraduate course, evaluating the graduate and the training body (Parliamentary Agenda for Responsible Health - Political Affairs Commission - CAP/CFM, 2018).

The national expansion of PT, in which most schools apply it without effective participation in the elaboration of items, weakens one of the main advantages of the program: the educational synergy in the network. This fragility, added to financing problems, especially for public schools, and the lack of adequate feedback after analyzing the results, essential in the PT, limit the PT training potential to improve medical education in the country. The interpretation of the results for necessary adjustments, both by schools and at the national level, needs to be taken seriously, because the absence of this process can configure a transformation of the PT into a serial MLE, focused only on cognitive aspects (TRONCON, 2022). 

The undergraduate evaluation processes need to consider, in addition to knowledge and skills, the professional identity formation, known as Professionalism. This concept is more complex and contextual, not just operational. It encompasses desirable behaviors and attitudes in medical practice, rather than a fixed set of attributes, such as a holistic ethos, aligned with humanism, which incorporates empathy for the patient, work-life balance, and integrity (BIRDEN, 2013). Although this set of attributes is difficult to evaluate, conceptual frameworks defined in different countries are sought, such as those of the Accreditation Council for Graduate Medical Education (ACGME), in the United States of America, and the Canadian Medical Education Directives for Specialists (CanMEDS) (CANADA, 2015; ACGME, 2024). 

The tools for the evaluation of Professionalism are many, and they are always implicated in the context of professional skills evaluation. In this context, Entrustable Professional Activities or EPAs have been gaining relevance as practical criteria for monitoring the acquisition of competencies (TAY, 2020). EPAs are defined as competence milestones, where the trainee is already able to perform, safely and reliably, without supervision, complex activities that denote mastery of a complete set of attributes. These EPAs are established in systematic, broad consultations with a group of professionals of a given profession or specialty (TEN CATE, 2005). The set of EPAs, properly certified by instructors, constitutes a flexible training track, capable of also involving aspects of the so-called parallel curriculum. 

If the 2000-2010 decade was considered worldwide the period of competency-based education (TEN CATE, 2005), in Brazil most schools remain with content-focused teaching. To advance in institutional evaluation, we first need to advance in curricular models and student evaluation processes, remembering that the latter are a driving force not only for progression, but also for learning (EPSTEIN, 2007).

 The global trend is the adoption of programmatic assessment, an approach that combines the view of complexity with the need to maintain an integrated and holistic evaluation. Rather than deconstructing competence into discrete, individually assessable units, programmatic assessment involves building a meaningful, global narrative about student performance, based on a triangulation of information from multiple sources, longitudinal data collection, meaningful feedback, and targeted learning activities. This approach requires clear and transparent justification for each decision of high importance and seeks to ensure the validity and quality of evaluations, through a system perspective, rather than isolated methods (SCHUWIRTH, 2020). To this end, the ideal would be the creation of an Assessment Board per school, capable of studying, developing, and applying the evaluation tools and sequencing the various stages of the student’s development in a specific dossier. The external evaluation, whether certifying or normative, would be based on these Boards’ audits, with pre-defined criteria. 

The impossibility of evaluating all these performance aspects in specific measures such as MLEs is reflected in their current formats, all based only on methods of assessing knowledge and skills. If the MLE has only written questions, its validity will be further compromised, as it excludes all practical competencies from the assessment (ARCHER et al, 2015).

To get closer to these objectives, both assessments should, at a minimum, include simulation scenarios, such as the Objective Structured Clinical Examination (OSCE) to assess clinical skills. This would level them with contemporary models of MLEs in range and limitations. Of course, this design would be expensive and laborious, requiring abundant inputs and human resources, which would decrease evaluations feasibility. However, considering the relevance of the objectives set, this greater investment is essential, as it is not possible to widely assess the aptitude of the thousands of students graduating annually in Medicine with resources of inferior technical quality (BICA & KORNIS, 2020).

On the other hand, this limitation does not justify maintaining the individual assessments of medical schools as a guarantee of quality in professional performance, since there is already clear evidence that the rigor of their evaluation systems is very variable, and the performance of their graduates in standardized external evaluations, also significantly so (MCMANUS et al, 2008; MCCRORIE & BOURSICOT, 2009). 

Thus, when taken together, these analyses do not allow inferences about the quality of the evaluations of individual medical schools. However, they show that the qualifications of recent graduates are different, even in countries with a small and stable number of medical schools: doctors with distinct levels of aptitude receive the same degree, with the same attributions and responsibilities. In a country like Brazil, with a much greater variability in education quality, this distortion is certainly more serious and cannot be ignored (BICA & KORNIS, 2020).

Parallel to this national context, there is a large number of Brazilians studying in medical courses in other Latin American countries, among which stand out Bolivia, Cuba, Paraguay, and Argentina, stimulated mainly by lower tuition fees and who, annually, have enrolled in the process of revalidation of their diplomas so as to practice in Brazil. 

For a doctor to work in another country, it is necessary to revalidate the medical diploma. This process varies, as countries are free to create their own revalidation rules. Before 2011, this revalidation was an individualized function of public universities. From then on, Brazil aligned itself with developed nations by establishing the National Exam for the Revalidation of Medical Diplomas Issued by Foreign Higher Education Institutions (REVALIDA), through Interministerial Ordinance MEC/MS No. 278, of March 17, 2011 (BRASIL - MEC, 2011), an exam with no interference from medical entities. REVALIDA consists of a two-phase knowledge test (theoretical and practical), representing the signatory public universities, with the objective of verifying the acquisition of knowledge, skills, and competencies required for professional practice appropriate to the principles and needs of SUS at a level equivalent to that required of physicians trained in Brazil. Thus, the idea was to use appropriate isonomic parameters and criteria to assess curricular equivalence and define the corresponding aptitude for the professional practice of medicine in Brazil. 

Importantly, the quality of medical education, whether undergraduate in Brazil or abroad, should not be neglected in favor of mass graduation. 

The results of this scenario of many medical graduates in Brazil and in the surrounding countries will be deeply felt, both in the labor market and in the quality of medical care in the coming generations, in view of the well-known professional longevity of doctors. Certainly, the economic and social cost of many doctors with deficient professional training will have dire consequences on health indicators, with an increase in the request for unnecessary complementary tests, diagnostic errors, iatrogenesis, excessive medicalization, among others. All without the guarantee of an adequate distribution of doctors in the various regions of the country, including the most remote areas.

The existing or already used evaluations in the national scenario are in the following situation:

1) The National Student Performance Exam (ENADE), which is triennial, has shown high grades in courses with a known dubious quality. This has occurred since most schools have held preparatory courses with students in the ENADE year of the health area or have enrolled only the best students in the evaluation, causing evident result biases. On the other hand, institutions with low ENADE scores maintain extensive misleading propaganda in the several types of media.
2) National Serial Assessment of Medical Students (ANASEM), proposed for different annual assessments for the 2nd, 4th, and 6th years. Even though it was created by Law (No. 12,871, of 2013) and instituted by MEC Ordinance No. 982, of August 25, 2016, it was applied only once in the second semester of 2016, for students in the second year. The result did not discriminate against the quality of the training of the courses, since more than 92% had an adequate evaluation. This assessment did not take place the following year, being extinguished by Law 13,530 of December 7, 2017.
3) The National Exam for the Revalidation of Medical Diplomas Issued by Foreign Higher Education Institutions (REVALIDA) has been applied annually since 2011. Due to many legal appeals, the 2018 and 2019 editions were not held, and it was applied again in 2020, when it started to be applied every six months (BRASIL, 2019). It has been applied every four months since 2023 (BRASIL, 2023).

There is a moment of complete uncertainty about the directions to be followed regarding the evaluations of medical students, even in the face of existing experiences (ENADE, ANASEM and REVALIDA) and, certainly, there are many economic and political pressures for these evaluations to be just a rite of passage. Regardless, the Brazilian society cannot renounce an adequate evaluation of medical training.

On the other hand, there is no sense in conducting three evaluations for the same purpose, in view of all the technical, logistical, and budget difficulties to ensure an adequate verification of the quality of the medical professional being trained.

It is essential to address these issues raised about the obligation to evaluate training quality of the physicians to practice in Brazil, whether they are trained in the country or not, without risking an exaggerated simplification for an evaluation that is known to be complex.

A round table at the 58th Congress of the Brazilian Association of Medical Education in 2020 (online), with the presence of the entire board and presidency of the National Institute of Educational Studies and Research (INEP), discussed the proposal of a single evaluation that meets all the same objectives or, in view of the political and legal difficulty for this to occur, that ANASEM would become a more summative progress test, unified with the ENADE of medical courses and REVALIDA, so that students from Brazilian medical schools in the second, fourth, and sixth years would be submitted to the same theoretical (objective) test as in the first phase of REVALIDA. Such an application would characterize an annual national progress test for Brazilian students. This way, each student would take three theoretical exams throughout their medical training. This would allow the situational diagnosis of the national evaluation of medical courses, according to the type of institution and country region, with the establishment of an individual historical series, both of students and of public and private institutions. The second phase of REVALIDA, conducted through simulated practical stations, would be done for Brazilian graduates (sixth year) and doctors trained abroad approved in the first phase of REVALIDA. Thus, the allegation that Brazilian and foreign doctors trained abroad are being subjected to a more difficult evaluation would be dismissed. Thus, there would be the defense of the greatest national interest, which is the assistance of the population by qualified doctors, which could contribute to the improvement of SUS. 

The result of this joint evaluation (theoretical and practical) for Brazilian sixth-year students and physicians trained abroad could represent a national medical residency exam for the programs that accepted such a proposal, and those selected would occupy the vacancies according to their preference options. This would also make this selection process more transparent, which is also very necessary.

Based on the assumption that the result of the evaluation of Brazilian students should make the students themselves and their undergraduate courses co-responsible for the adequacy of medical training, it will be necessary to create criteria for good and bad results of this joint evaluation, with sanctions for both parties (students and courses) and provision for remedies.

This issue, although urgent, is far from a resolution, as it depends on the political articulation of the actors involved (academic community, medical corporations, and representatives of the Legislative and Executive Branches), the strength of social demands on the subject, and the balance between government priorities for the expansion of medical education and regulation of its quality. There has been no collaborative effort to build an evaluation model that is minimally acceptable to all, which would require concessions from stakeholders.

A new Bill (LB 2.294/2024) proposes that the practice of medicine in Brazil requires the approval of a proficiency exam. According to the project, only after the exam will graduates in the area be able to register with the Regional Council of Medicine. According to the author, this demand is from the entities representing the profession themselves, which points to the indiscriminate proliferation of courses in the country and the inferior quality of training, that is, the same justification of the old bills that were not approved. The proposal for the National Proficiency Exam in Medicine, which is supported by the Federal Council of Medicine, provides for its offer, at least twice a year, in all states and the Federal District.

Thus, there is an urgent need for an evaluation process that involves students and medical schools, so that they fulfill their respective roles with the commitment to the quality of medical education throughout the undergraduate course. 

The serial evaluation process has been considered the best alternative. In view of the complexity of applying it to all medical courses in the country, some discussion fronts have proposed a terminal evaluation, which would take less time to implement. The proposal of the National Proficiency Exam (ENP), supported by the Federal Council of Medicine, provides for its offer, at least twice a year, in all states and in the Federal District. Thus, although technically it is not the best option, it is an acceptable start to have an evaluation of medical training in Brazil and to be able to advance in this urgent need to change the current situation. In addition, it would be a way to evaluate those approved in the ENP so that they could, from the exam, request their registration in the CRM and be legitimized and authorized to practice the medical profession. It would constitute an evaluation of a national character, made by an institution unrelated to the reality of each higher education institution (HEI), independent, and, therefore, exempt and without any built-in conflict of interest. An important discussion will be about who will prepare this type of evaluation and what will be its consequences, especially for HEIs that are notable for being deficient in their medical courses’ training.

It is useless for courses to have excellent physical infrastructure of classrooms, different laboratories with the use of high technology, qualified faculty, and psychopedagogical support centers for students and teachers, indicators that lead to grade 5 in INEP/MEC, if students do not have a good ethical and professional training, emotional balance, and adequate mental health to start and maintain their medical career. And this needs to be objectively reflected in professional training evaluations that are not only those conducted in the medical course itself.
